# Mapping health behaviour related to Chagas diagnosis in a non-endemic country: Application of Andersen’s Behavioural Model

**DOI:** 10.1371/journal.pone.0262772

**Published:** 2022-01-20

**Authors:** Laura Iglesias-Rus, María Romay-Barja, Teresa Boquete, Agustín Benito, Briggitte Jordan, Teresa Blasco-Hernández

**Affiliations:** 1 Centro Nacional de Medicina Tropical, Instituto de Salud Carlos III, Madrid, Spain; 2 Red de Investigación Colaborativa en Enfermedades Tropicales, RICET, Madrid, Spain; 3 Fundación Mundo Sano, Madrid, Spain; National Institute for Infectious Diseases IRCCS Lazzaro Spallanzani, ITALY

## Abstract

**Background:**

Chagas disease has become a challenge for non-endemic countries since population mobility has increased in recent years and it has spread to these regions. In order to prevent vertical transmission and improve the prognosis of the disease, it is important to make an early diagnosis. And to develop strategies that improve access to diagnosis, it is important to know the factors that most influence the decision of the population to know their serological status. For this reason, this study uses Andersen’s Behavioural Model and its proposed strategies to explore the health behaviours of Bolivian population.

**Methods:**

Twenty-three interviews, two focus groups, and two triangular groups were performed with Bolivian men and women, involving a total of 39 participants. In addition, four interviews were conducted with key informants in contact with Bolivian population to delve into possible strategies to improve the Chagas diagnosis.

**Results:**

The most relevant facts for the decision to being diagnosed pointed out by participants were having relatives who were sick or deceased from Chagas disease or, for men, having their pregnant wife with a positive result. After living in Spain more than ten years, population at risk no longer feels identified with their former rural origin and the vector. Moreover, their knowledge and awareness about diagnosis and treatment still remains low, especially in younger people. Limitations on access to healthcare professionals and services were also mentioned, and proposed strategies focused on eliminating these barriers and educating the population in preventive behaviours.

**Conclusions:**

Based on Andersen’s Behavioural Model, the results obtained regarding the factors that most influence the decision to carry out Chagas diagnosis provide information that could help to develop strategies to improve access to health services and modify health behaviours related to Chagas screening.

## Introduction

Chagas disease (CD) is a parasitic infection caused by *Trypanosoma Cruzi* (*T*. *cruzi*). The main route of transmission in endemic countries is through triatomine insects, although congenital transmission, solid organ transplants, and blood transfusions are also possible transmission routes both in endemic and non-endemic regions [1].

CD has an asymptomatic acute phase that lasts 4–8 weeks, during which parasitaemia levels are higher; then it enters an indeterminate chronic phase, without signs or symptoms of visceral involvement [1]. If CD remains untreated, parasites could produce gastrointestinal and/or cardiac damage, causing megaviscera or cardiomyopathy in 30%-40% of patients [1]. These complications of CD cost healthcare systems $627 million per year and lead to numerous disabilities [1].

Available treatments for CD are benznidazole and nifurtimox, both of which have side effects [1]. Current evidence shows that treating infected women before pregnancy reduces vertical transmission of *T*. *cruzi*, and the cure rate in congenitally infected new-borns is close to 100% [1–4]. However, treatment is less effective in patients in the indeterminate phase or with visceral involvement [1]. That is why early diagnosis helps eliminate the parasite and prevents cardiac and gastrointestinal damage [1–5].

CD is currently an endemic disease in 21 Latin American countries, usually in impoverished rural environments. However, migration from rural to urban areas within the same country and mobility to other non-endemic regions such as North America and Europe allows its spread to regions that were not previously affected [1–6]. Estimated prevalence of CD in Latin American population living in Europe is about 4.2%, where the highest burden is borne by Bolivian population (18.1%) [1–7]. Spain is the European region with the highest burden of CD, having an estimated 52,000 cases, and 81% of them reported; nonetheless, a cross-sectional survey carried out in Madrid about Chagas health-seeking behaviour showed that only 44% of Bolivian population living in Madrid know their serological status, which means an estimated prevalence of 27.7% [8–10]. However, other than ensuring safe blood transfusions and organ transplant since 2005 [11], no national strategies have been developed for screening the at-risk population that Spain receives annually from endemic regions. Despite evaluation studies pointing out Chagas screening as a cost-effective strategy in non-endemic areas [5, 6, 12, 13], few Spanish regions (Galicia, Valencia and Catalonia) developed protocols for primary care testing, and some health professionals published guidelines encouraging Chagas screening at primary care services [14].

CD is also a health problem with psycho-emotional and socio-anthropological aspects. It is frequently associated with death, misery, dirt and rural areas, and despite of its normalisation among Bolivian people it is an unease issue to talk about because of the fear of rejection and social isolation [15–20]. Moreover, women are afraid of passing the disease to their children and have a sense of guilt and responsibility in relation to this fear [15, 16, 18, 21]. All these elements along with misconceptions about the symptoms and the treatment make affected individuals hide the disease and sometimes they avoid knowing their serological status [15, 17, 18]. In addition, recent studies showed mental distress in patients with CD [22].

**Fig 1:**
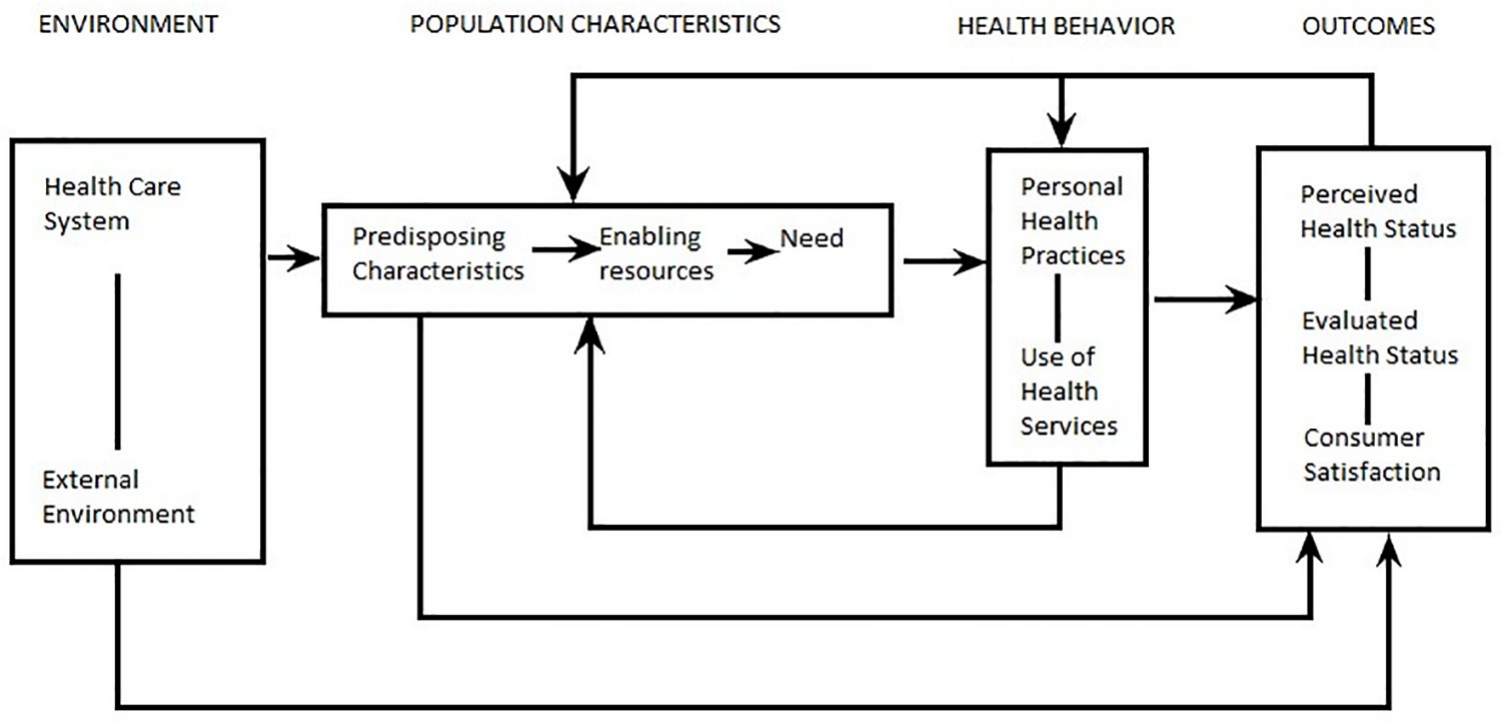
Andersen’s Behavioural Model of health service use [24].

Andersen’s Behavioural Model of Health Service Use allows us to understand, from a systemic perspective, in which way are health behaviours, health outcomes, and access to healthcare services influenced by patient and environmental factors [23]. This model evaluates the interaction between the characteristics of population, the healthcare environment, and external environmental factors [24]. Population characteristics include predisposing factors intrinsic to the patient such as age, sex, social structure, health beliefs, values, and cultural norms; enabling resources, which include those resources available in their closest environment (personal, family and community resources); and need, which is patient or health professional’s judgement on health status and medical care perceived. Health behaviour involves those actions being carried out to achieve health or well-being [24, 25] (Fig 1).

Andersen’s Behavioural Model has been broadly used in studies as a guide to examine health service use in different racial or ethnic populations [26, 27]. However, no studies with this theoretical framework in relation with Chagas diagnosis in non-endemic countries have been found. Since many people living with Chagas cannot afford the benefit of being diagnosed, a thorough understanding of their perceptions on the care provided and the factors that influence the search for their health could allow the development of adapted interventions that facilitate diagnosis [1]. This study assessed factors that influence Bolivian population’s health behaviour in relation to Chagas diagnosis according to Andersen’s Behavioural Model and its proposed strategies that could influence such behaviour aiming to provide information to develop focused interventions to improve access to screening.

## Methods

### Ethics statement

The study was approved by the Ethics Committee of the Spanish National Health Institute, Carlos III (reference: CEI PI 05_2015-v2). Written informed consent was obtained from all the participants in order to use and publish the findings. Personal data were managed according to Spanish legislation [28].

### Setting

The present study was conducted in Madrid, Spain, a non-endemic country for CD. According to the municipal census on 1 January 2017, a total of 15,951 Bolivians lived in Madrid, 6,758 of which were men, and 9,193 were women. This population is mainly distributed in the municipal districts of Usera, Carabanchel, Puente de Vallecas, and Latina [10].

The Spanish healthcare delivery system consists mainly of primary care and secondary and tertiary care, in health centres and hospitals, respectively. Health centres providing primary care in this region are distributed in seven areas of assistance from the organizational point of view, each one with its reference hospitals. However, population can be treated at any health centre or hospital due to current legislation [29].

Spain’s Health system was free of charge (public funding), but in 2012, access to public healthcare was linked to registration in the social security system, so access for undocumented migrants was limited to emergency services [30]; from 2018 onwards, it was necessary to prove a three-month stay in Spain in order to access to public healthcare [31]. Some hospitals and health centres offered the possibility of diagnosis and treatment despite of these limitations.

The most common test used for CD screening is ELISA because of its high sensitivity and specificity [1]; however, the results are known a few days later.

### Study design

This is a qualitative study with a phenomenological perspective comprised of focus groups (FGs), semi-structured interviews and triangular groups (TG) as the main data collection techniques. TG are composed of fewer people, have a less structured dynamic than FGs and merge the individual and the group discourse [32, 33]. A topic guide to ensure that interviewers got the necessary data to achieve the study’s aims was designed, which also allowed deeper exploration of the emerging topics. This study was part of a project to assess access and use of healthcare services among the Bolivian population in Madrid. Factors related to knowledge, attitudes, and practices of Bolivian population regarding management of CD, care itineraries, screening, and treatment were collected through a previous questionnaire [10]. This study presents the results of FGs, interviews and triangular groups performed with Bolivian population and key informants regarding factors influencing the diagnosis of Chagas.

### Participant recruitment

The sampling technique was convenience sampling according to the sampling types established by Teddlie and Yu [34]. Inclusion criteria were that the participants had to be over 18 years of age and have their residence in Madrid. The sample was stratified in Bolivian women with a positive diagnosis, Bolivian men with positive diagnosis and key informants in contact with Bolivian population resident in Madrid.

Interviewed Bolivian people were contacted in the Bolivian Consulate by the research team, and other participants were contacted through a health agent. Key informants were contacted through the network of the National Centre of Tropical Medicine. Participant recruitment was carried out until no relevant new information was obtained from additional interviews [i.e. the point of saturation was reached] [35].

### Data collection

The research team reviewed the topic guide for interviews, FGs, and TGs to ensure the potential to trigger a discussion and its comprehensibility following the Kvale model [36]. This guide was modified with the transcriptions of following interviews, FGs and TGs during data collection when finding topics that needed more details or inclusion of new questions (S1 File).

The topic guide included the following core themes: 1) knowledge about CD (transmission, symptoms, and treatment); 2) attitudes towards CD; and 3) practices in relation to CD (health behaviour).

Digital recorders were used during all the techniques. Furthermore, sociodemographic variables (sex, age, region of origin, education level, employment status, public health coverage, number of children, and years living in Spain) were collected as complementary information of the participants. These data were written down on paper and entered and codified into a spreadsheet to assure confidentiality afterwards (S2 and S3 Files).

### Data transcription, translation, and analysis

Audio files were transferred to a computer after recording. An external expert transcribed interviews, TGs, and FGs. Afterwards, a quality control of transcriptions was carried out by listening the recordings and correcting possible errors. Two researchers performed an inductive analysis of the topics based in Grounded Theory [37]. Initially, the transcripts were coded line by line following an inductive approach, creating emergent codes grouping the content of each sentence or paragraph. Codes and groups were further examined looking for thematic patterns in the data. The software MAXQDA 2018 (VERBI GmbH, Berlin, Germany) and Atlas.ti 7.0. Scientific Software Development 2009 (Gmbh, Berlin, Germany) were used to facilitate the coding process. Thematic analysis of FGs, TG and interviews was performed separately by the researchers involved in analysis. Joint meetings of those researchers were held to combine analysis results, and to carry out discussion about data collection and analysis procedures. Data sources and analysis triangulations were performed to provide concrete findings. All the gathered information was compared from different points of view and verified by the researchers. Table 1 shows categories and codes established in the analysis.

**Table 1:** Categories and codes established in the analysis.

**Diagnosis**	**Knowledge**	**Attitudes**	**Strategies to facilitate diagnosis**
Reason for diagnosis	Disease	Perceptions about the disease	
	Transmission	Perceptions about the treatment	
Place of diagnosis	Diagnosis	Stigma	
	Treatment	Perceptions about barriers of access	
	Healthcare delivery system		

After reviewing the domains and associated definitions of Andersen’s behavioural model, the researchers mapped topics and subtopics to each domain.

## Results

### Characteristics of the study population

Fifteen interviews, two TG and two FG with women were performed between the years 2015 and 2016; and eight interviews with men were performed between the years 2017 and 2018, involving a total of 39 participants. Moreover, four interviews were developed with key informants in contact with Bolivian population between the years 2017 and 2018 in order to know more deeply the most appropriate strategies to improve diagnosis in a non-endemic country. Tables 2 and 3 present sociodemographic characteristics.

**Table 2:** Sociodemographic data of Bolivian population.

	Participant code	Sex	Age	Region of origin	Education level	Employment status	Public Health coverage	Number of children	Years living in Spain
Interview	E1MP	Woman	31	Cochabamba	Secondary education	Housekeeper	Yes	2	10
	E2MP	Woman	33	Santa Cruz	Primary education	Housekeeper	Yes	3	10
	E3MP	Woman	45	Cochabamba	Primary education	Housekeeper	Yes	4	9
	E4MP	Woman	42	Vallegrande/Santa Cruz	Primary education	Housekeeper	Yes	3	7
	E5MP	Woman	34	Cochabamba/Santa Cruz	Secondary education	Housekeeper	Yes	3	9
	E6MP	Woman	44	Santa Cruz	Secondary education	Beautician	Yes	1	13
	E7MP	Woman	31	Cochabamba	Secondary education	Housekeeper	Yes	2	10
	E8MP	Woman	35	Santa Cruz	Primary education	Housekeeper	Yes	3	9
	E9MDD	Woman	40	La Paz	Universitary education	Civil servant	Yes	0	6
	E10MP	Woman	34	Cochabamba	Secondary education	Housekeeper	Yes	1	15
	E11MP	Woman	47	Cochabamba	Secondary education	Housekeeper	Yes	3	9
	E12MP	Woman	28	Santa Cruz	Secondary education	Housekeeper	Yes	1	6
	E13MP	Woman	47	Cochabamba	Secondary education	Housekeeper	Yes	3	9
	E14MP	Woman	50	Trinidad/ Santa Cruz	Secondary education	Unemployed	Yes	5	9
	E15MP	Woman	53	Santa Cruz	Secondary education	Cleaning worker	Yes	3	16
	E16HP	Man	35	La Paz	Secondary education	Housekeeper	Yes	2	12
	E17HP	Man	28	Cochabamba	Universitary education	Civil servant	No (private coverage)	0	6
	E18HP	Man	47	Tarija	Secondary education	Craft and related trades workers	No	3	11
	E19HP	Man	48	Cochabamba	Primary education	Craft and related trades workers	Yes	3	15
	E20HP	Man	47	Cochabamba	Primary education	Housekeeper	Yes	5	10
	E21HDD	Man	29	Santa Cruz	Secondary education	Housekeeper	Yes	0	14
	E22HP	Man	46	Cochabamba	Primary education	Craft and related trades workers	Yes	3	25
	E23HP	Man	52	Cochabamba	Secondary education	Craft and related trades workers	Yes	3	18
Triangular groups	TG1MP	Woman	31	Cochabamba	Secondary education	Housekeeper	Yes	2	10
		Woman	34	Cochabamba	Secondary education	Housekeeper	Yes	1	15
		Woman	47	Cochabamba	Secondary education	Housekeeper	Yes	3	9
	TG2MP	Woman	28	Santa Cruz	Secondary education	Housekeeper	Yes	1	6
		Woman	31	Cochabamba	Secondary education	Housekeeper	Yes	2	10
		Woman	31	Cochabamba	Secondary education	Unemployed	Yes	2	10
Focus group	FG1MP	Woman	44	Santa Cruz	Secondary education	Housekeeper	Yes	1	15
		Woman	38	Santa Cruz	Primary education	Housekeeper	Yes	3	9
		Woman	59	Santa Cruz	Secondary education	Unemployed	Yes	2	18
		Woman	50	Trinidad	Secondary education	Housekeeper	Yes	5	14
		Woman	46	Santa Cruz	Secondary education	Housekeeper	Yes	2	15
		Woman	47	Santa Cruz	Secondary education	Housekeeper	Yes	3	16
	FG2MP	Woman	30	Santa Cruz	Secondary education	Health agent	Yes	1	12
		Woman	31	Cochabamba	Secondary education	Unemployed	Yes	1	9
		Woman	42	Sucre	Universitary education	Unemployed	Yes	3	10
		Woman	56	Santa Cruz	Secondary education	Housekeeper	Yes	1	14

MP: Woman with positive diagnosis HP: Man with positive diagnosis MDD: Woman undiagnosed HDD: Man undiagnosed

**Table 3:** Sociodemographic data of key informants.

		Sex	Age	Region of origin	Education level	
Interview	E1RC	Man	50	Cochabamba	Universitary education	Journalist
	E2RC	Woman	30	La Paz	Universitary education	Health agent
	E3RC	Woman	38	Santa Cruz	Secondary education	Health agent
	E4RC	Man	40	Spain	Universitary education	Health program coordinator

Thirty women (97.8%) and seven men (87.5%) had a positive test for CD, whereas two participants did not know their serological status. Average age of participants was 40.3 years, aged between 28 and 59 years old. Most of them were women (79.5%), and 20.5% of them were men. Only two men (5.1% of interviewed Bolivian population) had no public health coverage. Regarding their level of education, most of the participants in our sample reported having completed secondary education and beyond (79.5%). Most participants reported having children (92.3%), and their average time living in Spain was of 10.3 years.

With respect to Bolivian population’s health behaviour in relation to CD, interviewed participants discussed fifteen factors that influenced CD diagnosis: six of them related to predisposing characteristics (age, work conditions, migration status, knowledge and health beliefs about CD and its treatment, stigma and shame associated with CD, and culture and health), four in relation with enabling resources (family history of CD, social and/or familiar support and advice, and accessibility to health services and knowledge about health system), three factors related to need (symptoms, attitudes, and subjective assessment about health) and two related to healthcare environment (system factors, and provider factors) (Table 4). Finally, strategies to facilitate diagnosis were also discussed.

**Table 4:** Themes and subthemes according to Andersen’s Health Behavioural Model.

POPULATION CHARACTERISTICS	Predisposing characteristics
	• Sociodemographic factors: age, work conditions, migration status
	• Knowledge and health beliefs about CD and its treatment
	• Stigma and shame associated with CD
	• Culture and health
	Enabling resources
	• Family history of CD
	• Social and/or family support and advice
	• Accessibility to health services
	• Knowledge about the health system
	Need
	• Symptoms
	• Attitudes
	• Subjective assessment of health
HEALTHCARE ENVIRONMENT	• System factors
	• Provider factors

### Population characteristics

#### Predisposing characteristics

Predisposing characteristics are those factors intrinsic to the patient. Bolivian people interviewed identified six factors: age, work conditions, migration status, knowledge and health beliefs about CD and its treatment, stigma and shame associated with CD, and culture and health.

Among all these factors, stigma and shame were frequently mentioned by older participants with some differences between men and women. Men explained that CD is associated with poverty and rural areas, so there is a fear and reject of being identified as poor peasants, and for this reason they avoid knowing their diagnosis. On the other hand, women commented their concern about rejection of the Spanish population due to ignorance about contagion and its possible consequences, such as job loss or social isolation. Consequently, both men and women tend to talk about CD only with their relatives and their closest social environment.

Interviewed population also described Latin American people’s culture as quite careless in terms of health, so they focus on issues that they consider more important, and health care is postponed. Thus, men reported the economic maintenance of the family as a priority, whereas women add to this objective the care of the family.

Regarding age, older participants pointed out that young people are more carefree about CD, explaining that they are not concerned about health at that age and they do not feel identified with endemic environments because many of them were very young when they came to Spain, and have always resided in the city.

Regarding knowledge and health beliefs about CD, participants expressed that their knowledge is very low, and it is based on their own experiences, although women pointed out that a positive diagnosis triggered a desire for new information about it. Furthermore, interviews and groups composed by younger men and women reported having less knowledge than older participants. Interviewed people are aware that CD affects a large part of Bolivian population due to the presence of vinchucas in rural areas. In consequence, they believe that since they have been away for a long time or have had no contact with them, they are no longer possible carriers of the parasite. Moreover, participants expressed confusion about vertical transmission. They assume CD is hereditary from both parents but cannot explain why some children are born without the disease in Spain or Bolivia. Regarding symptoms, interviewed people clearly identified heart problems and sudden death in people at an older age, and they also consider the possibility of not having manifestations of the disease throughout their life. In addition, they explained that a positive diagnosis already implies that there is a disease, and this knowledge could deteriorate their health, and that is why they avoid performing the diagnostic test. Finally, participants showed doubts about the efficacy of CD treatment and were afraid of its serious side effects, so they consider that it makes no sense to know they have Chagas if they have no chance of being cured.

Both men and women also mentioned that despite having a health insurance card, their work conditions (such as schedules or impediments from their bosses) limit their attendance to medical appointments, since they fear that absence from their job or the perception of being ill may cause them to lose their job. As an alternative, men explained that using private healthcare is more compatible with their schedules. Furthermore, interviewed people reported the experiences of their social environment regarding the difficulties obtaining healthcare as immigrants; due to the bureaucracy to achieve it, their use of resources is limited to acute and emergency situations. Table 5 shows the quotes related to predisposing characteristics.

**Table 5:** Quotes related to predisposing characteristics.

Age	“When you are young you think the world is in the palm of your hands and everything else does not interest you, let’s say, right? Moreover, the young person is more…they want to be alive, to go out, to dance, I don’t know…to travel, to have fun (…) That is young people’s mindset, I think.” (Woman, Interview 9)
	“I told to my son ‘get the (Chagas) test’, ‘No, mum (…) How will I have that (disease)?’” (Woman, FG 1)
Work conditions	“Here they do not do it (Chagas test) because, perhaps, they are working, and they have to ask for permission. . . they do not get permission! Now, I took the opportunity to come because I have the morning off, but (…) if I have a lunchtime break, I use it to go to the doctor at lunchtime, for example.” (Woman, Interview 6)
	“I don’t go to the doctor because I have no time. Because I am self-employed (. . .) I hardly ever go to the doctor. If I go… I go at night because I have a private doctor.” (Man, Interview 19)
	“There are few (immigrants) who work in an office (…). Most are bartenders, caregivers of adults and children, housekeepers…so their salaries do not reach a thousand euros, right? (…) I think all the immigrants sometimes have two jobs in order to meet their needs, so it is another factor, right?” (Woman, Interview 9)
Migration status	"And now people who don’t have residency papers (…)they directly do not have a health insurance card” (Woman, Interview 5)
	“Last year, they brought out a law that they didn’t have a right (of health care)…that people without a residence permit (…) I got a bill for an emergency care for my daughter.” (Woman, Interview 12)
Knowledge and health beliefs about CD and its treatment	“I really wasn’t fully aware of that disease (…) I didn’t really know what it was about (…) The doctor at the hospital told me about it(…) He sent me to Tropical Medicine (…) and there they explained to me what it was. Well…the concern began to affect me there.” (Woman, interview 1)
	‴And how can it be if you didn’t live there, in the countryside?’ (…) I didn’t live in those houses in Bolivia (…) I think that most of them have it because they are very poor people who live in the countryside, so…” (Woman, Interview 8)
	"At first, they thought it was hereditary because my older sister had it, my second sister had it (…) they said ‘We all assuredly have it. It is hereditary’ Then the youngest sister…she didn’t have it!” (Woman, Interview 5)
	"So, what I have always heard is that Chagas disease manifests in twenty years’ time. (…) That’s what people thought, right?" (Woman, Triangular group 2)
	"…as it is a silent disease, you feel fine (…) people can die of old age but…they might have had Chagas and they had not even found out.” (Woman, Interview 8)
	"But… it is said that over time you have problems, worries…all those things. It is said that virus progresses faster.” (Woman, Interview 5)
	"He told me that there was a treatment to keep it quiet, to keep it…you know, without manifesting (…) but it is not a cure. He said there was a cure for children, but not for the older people.” (Woman, Interview 10)
	"If there is no cure, why are you going to grieve, right? You will die anyway, sooner or later.” (Woman, FG 2)
Stigma and shame associated with CD	“I think there are people that even they know they have Chagas they do not want to get the test because they know it is going to be positive…because Chagas is known as a disease of the poor (…) So that would be a difficulty of access, cause of the shame, let’s say, right?” (Man, Interview 20)
	“I noticed in my work…my boss’s daughter (…) she wasn’t well informed about this, she said ‘Dad, are you sure this is not contagious?’ So, I felt like…like I was in that house and I could infect them, right?” (Woman, Interview 5)
	“I haven’t told my (social) environment, nobody knows, just my nieces. I live with two nieces, only they know it. Also, my friend, the one I usually hang out with, but no one else.” (Woman, FG 2)
Culture and health	“Or because of the fact that…people who are careless like us (about health)… so you leave it be (…) and you worry about other issues and then you don’t do what you have to do (about health care).” (Woman, Interview 2)

#### Enabling resources

Enabling resources are tools or resources that facilitate healthcare use. Participants in this study identified four factors: family history of CD, social and/or family support and advice, accessibility to health services, and knowledge about the health system.

Family history of CD helped interviewed people with the decision of Chagas diagnosis. They explained that having a relative with a positive diagnosis and onset of symptoms or decease at certain age made them think about the possibility of getting diagnosed when reaching that age, unlike those with asymptomatic, positive relatives in good health or dead from causes other than CD. Specifically, men reported that after the positive diagnosis of their wives during pregnancy follow-up they decided to know their serological status. With respect to children, they waver between doing the study after one of the parents is diagnosed or waiting until they are adults, because they consider that until then the disease may not affect them. On the other hand, those with no family history considered that it was not necessary to know if they were bearers of Chagas.

In addition, participants pointed out that it was easier for them to make the decision to go to a health centre for the diagnostic test if a family member or a friend recommended it.

Regarding health services in Spain, women and men mentioned that the possibility to get health care is limited by geographic distance from the Tropical Medicine unit, explaining they sometimes deterred from keeping appointments because of the time spent in public transport from their workplace.

Finally, participants, especially men, referred their lack of knowledge about the Spanish health system and their rights as users, explaining that they do not always know where the test can be performed apart from the hospital units where the disease is monitored, which services they can access as users of the health system, and whether these services may change due to their administrative situation (unemployment or expiration of health insurance card).

Table 6 shows the quotes related to enabling resources.

**Table 6:** Quotes related to enabling resources.

Family history of CD	"My father died of a heart attack, my brother also, my sister just died of a heart attack. So, it worried me a lot, and I thought ‘What a shame to lose my sister so young’ (…) I got the tests, and fifteen days later they called me (…) I felt terrible (…) they told me I had Chagas disease.” (Woman, FG 1)
	“Because my mother lived almost 88 years, but if she had the disease, she would have died earlier, right?. . .And my grandmother lived until 115 years old.” (Man, Interview 19)
	“When my son was born, they tested her (his wife). And when they did the test (…) it came out that she had the problem (Chagas disease). (…) They tested me as well, I also resulted positive.” (Man, Interview 16)
	“My son says ‘No, no, I don’t want to know (…)’, and they are still young (…) now it is not time for them to know, and on the other hand it is said that Chagas affects from 35 years of age, so I do not know if it affects you when you are 15 or 20 years old…” (Man, Interview 20)
	“Because basically my whole family lives in La Paz, they are from La Paz, and no one has showed signs of that disease yet. (…) Because they have always been from La Paz. (…) from the city, so…Yeah, I could get the test (for CD), but I don’t believe I have it…” (Woman, Interview 9)
Social and/or family support and advice	“My mother told me ‘Why don’t you get the (Chagas) test done, so you can know it?’ Because my mother really wanted to know. Right… finally they told me it was positive.” (Woman, Interview 12)
	“Because my friend (…) She told me ‘Look, they detected Chagas to my mother, why don’t you get tested?’ And because it is my friend, I said ‘Ok, give me the address’” (Woman, FG 1)
Accessibility to health services	“When I had to go to that hospital it took almost an hour of commute. (…) And I have few (economic) resources right now (…) I work only two hours, and I know that it requires time, but it’s impossible for me. (…) I changed to another hospital, which is closer for me.” (Woman, FG 1)
Knowledge about the health system	“Perhaps the lack of information (…) that they can be tested in any public hospital (…) I don’t know, here in Spain or Madrid.” (Man, Interview 17)
	"I would like to know if (…) well, if they would continue to assist me (. . .) because I collect unemployment benefit, so if it ends (…) My doubt is if I will be attended as I have been attended so far, if I am not collecting the unemployment benefit." (Woman, Interview 1)

**Need.** Need is the patient or professional’s judgement about the requirements of health care. Interviewed participants identified three factors: symptoms, attitudes about CD, and subjective assessment of health.

Interviewed people expressed that the lack of symptoms before diagnosis made them feel less worried about CD, and they explained that feeling well is not a reason to use health services, except for activities such as donating blood, where some of them accidentally discovered their serological status. In addition, when symptoms such as fatigue or tiredness appear, they scribe them to work, daily life, or other different illnesses. However, participants explained that once they are diagnosed or know their family history of CD if they have signs of symptoms of any kind, they think it is because of CD complications and consult a physician.

On the other hand, older men and women expressed that CD is a deadly health problem, and this makes them feel uncomfortable, fearful, and unease. They frequently think about the possibility of being ill, and leaving their family due to an early death, or being limited for work; however, they would rather not know if they are sick so they do not to have to deal with it. In addition, they explain that knowing that it is a very common disease in Bolivia gives them peace of mind.

Finally, women worry about the effect that the disease may have on their children and they decide to have them diagnosed after knowing their own serological status. However, women who were diagnosed with Chagas during pregnancy had not considered this option before. They explained that health professionals did the screening as part of the pregnancy monitoring.

Table 7 shows the quotes related to need.

**Table 7:** Quotes related to need.

Symptoms	“Well, I think here (in Spain), and there (in Bolivia), it’s not a disease that, you know, has very strong symptoms, or is very noticeable, that is, that you know you have Chagas, for example. (…) It doesn’t manifest so you know at first sight that…so you notice….” (Woman, Interview 9)
	“It was in the last (blood) donation when they told me I could not be a blood donor… I still have the document (…) ‘Because you have Chagas disease’, they said. (…)” (Woman, Interview 11) "Some days I feel tired (…) Really tired (…) I start driving, and I feel tired. Of course, driving is not a hard task, it is only the sight, but…the body gets tired.” (Man, Interview 19)
	"I read that it affected the intestines and all that…now I can’t drink coffee, coffee makes me ill (…) I don’t know, maybe it’s because I have that disease…” (Woman, Interview 2)
Attitudes	“Maybe I’m going to die, I’m going to leave my daughters (…) I’m very worried ((cries)) I don’t know what to do… sometimes I think I’m going to die anytime.” (Woman, Interview 3)
	“And I have told my friends who are from my country and they don’t know if they have it (Chagas disease) or not, they have never been tested (…) Because they don’t want to know (…) Because they say they’d feel uneasy knowing they have it, and if they die anytime, it doesn’t matter. Because they don’t want to suffer for knowing they have it. That’s the reason.” (Woman, FG 2)
	“We do not classify the disease as serious. If you have it, that’s it, right? The bug has bitten you, or whatever, so we were not aware of the severity of the disease. And since there are many people that have it (…) sometimes one says ‘everyone has it, so…’ it calms you down, right? Sometimes that happens. Because in Bolivia there are many people who have it.” (Woman, FG 2)
	“How is it possible that we are going to have children who will be born with a disease that makes you die young?” (Woman, FG 2)
Subjective assessment of health	“My children are really worried (about me). They began to call me when they knew about Chagas. ‘Take care, mom’, because we recently lost my sister (…) What I said was ‘Ok, if you love me, get tested. Come on.’ (Woman, FG 1)
	“And now that… that I am pregnant again is when I have discovered… well, they told me about. . . they had requested this test (for Chagas). I think it was the first time I had been tested for this”. (Woman, Interview 10)

### Healthcare environment

#### System factors

System factors include those services that are complementary to patient care.

Participants explained that the limited flexibility of attention schedules and the extended waiting times at health centres and hospitals were inadmissible, and it is frequently confronted with other family and work responsibilities. For these reasons, they are frequently forced to leave the health centre without being treated, or they do not even request health care because they consider it a waste of time.

Table 8 shows the quotes related to system factors.

**Table 8:** Quotes related to system factors.

System factors	“The doctor knows that I leave work in a hurry, that I asked for permission, so I frequently complain ‘I have been waiting for you for 45 minutes’” (Woman, TG 1)
	“I’m afraid of being fired for asking permission to go to the doctor. I prefer to go to work than go to the doctor and waste time.” (Man, Interview 16)

#### Provider factors

Interviewed population commented that trust, professional knowledge, communication, and previous experiences with care providers influenced in CD diagnosis.

Participants consider that CD is not known by all the Spanish healthcare providers, explaining that when they consulted on the diagnosis or the management of CD, most physicians in primary care centres showed little knowledge about it; in fact, both men and women commented that it would be important for them if a physician recommended being tested. However, they value positively their reception and monitoring in the Tropical Medicine Units of the hospitals, pointing out that professionals there are more familiar with the disease.

In addition, participants pointed out that they have problems communicating effectively with physicians, since the time of care in doctor visits is very short, and they feel that the health problem they are reporting is approached superficially; therefore, they prefer to change physicians or not going back. Specifically, women explained that doctors did not seem to trust them when they reported about their family or personal history of CD, and they were required to prove it through clinical reports that were not always available.

Finally, interviewed population shared different negative experiences related with the attitude and behaviours of healthcare providers that made them feel discriminated and displaced as immigrants; for these reasons they avoid making appointments with the same professionals in order to avoid feeling bad in a psychological level.

Table 9 shows the quotes related to provider factors.

**Table 9:** Quotes related to provider factors.

Provider factors	“I think most physicians have no knowledge about…tropical diseases (…) Because they have requested blood tests for everything for me, and other things (supplementary tests), but they have never told me ‘go to get this test (for Chagas disease)’, despite the fact that my clinical history says I have it. They never say ‘Let’s see if it is true that you have it.’ Never. (…) In health centres nobody talks about it (…) they registered that I have it, but nothing else.’ (Woman, FG 2)
	“They don’t ask you if you are from Bolivia, but rather ‘Are you from Latin America? (…) Well, you have to get tested for Chagas disease’, I wouldn’t feel bad (…), and people would be grateful (…) ‘Oh, look, they care about me’, and if it has no cost for me, I will do it.” (Woman, Interview 15)
	“As soon as you arrive there (hospital), there is the Tropical Diseases area (…) When you are there, they are looking out for you (…) I love that, that’s why I have no problem, because they are attentive. . .” (Woman, FG 1)
	“I think that is the problem. We do not understand something, and we leave it be. If they don’t explain things to us correctly (…) they tell you things like that, and they think I must have understood. (…) But then, over time, I have realized that if you don’t request it, if you don’t ask ‘Look, I don’t understand, I need you to explain it correctly, I have the right to know what (disease) I have, what is the treatment, everything, until I understand it…” (Woman, Interview 12)
	“That happened to me with my family doctor (…) I told him ‘I have a positive result for Chagas disease’. ‘Ok, then you have to bring out the medical report, then I read it and I will tell you what treatment you need to take. So, you come to tell me what is going on.’ (…) ‘I can’t do anything until you bring me that report’.” (Woman, FG 2)
	“He (the family physician) ignored us. (…) ‘You have to go back to your country’ (…) You feel bad and you don’t go back to the doctor.” (Man, Interview 20)

### Strategies to facilitate diagnosis

Interviewed participants identified few strategies that could make them want to know their Chagas diagnosis, and they were mainly oriented towards mandatory screening for the Bolivian population or specific consultations for young adults in order to reduce waiting times.

On the other hand, key informants pointed out that testimonials from people diagnosed with CD may be useful to reinforce reasons for getting tested. Moreover, with the aim of reducing waiting times and shorten distances between population and health resources, they recommended designing events in places and environments close to the population (such as their neighbourhoods or traditional festivals) providing information as well as the opportunity to be tested simultaneously.

Other possibility they mentioned was to include the serological test in routine analysis, as well as to gather all medical appointments and tests to facilitate care.

Furthermore, key informants explained the importance of using messages focused on diagnosis when there are no symptoms to prevent cardiac and/or intestinal damage, and to educate women on childbearing age in order to prevent the transmission of the parasite to their children. However, they consider that it could be harmful to carry out campaigns in high schools, because it could give rise to the stigmatization of younger children by associating the disease to people from Latin America.

Table 10 shows the quotes related to strategies to facilitate diagnosis.

**Table 10:** Quotes related to strategies to facilitate diagnosis.

Strategies to facilitate diagnosis	“What we used to do was to look for spaces to inform through talks, and our main objective was looking places, centres where people would become aware and realize that the Chagas test matters (…) and take the next step, which is to go to hospital and get tested (…) Now, since 2017 (…) we give general information about Chagas disease (…) Then they go to a nurse and a blood sample is taken.” (Key informant, Interview 3)
	“It would be better (…) From 55 years of age, having a health centre just for them (…) They can wait as long as they need to (…) but us young people have limited time.” (Man, Interview 19)
	“It is necessary…not only an awareness raising campaign through media and social networks, there has to be information an action. (. . .) In Chagas day or whatever, doing a social, cultural or sport activity (…) in that moment do the testing (…) A connection between information, awareness, and action. (…) And using testimonies of those affected, right? ‘I was ill, I just noticed, I got cured, and now I want to share my experience.’” (Key informant, Interview 1)
	“He understood there is no cure for it. (…) In the end I told what happens with Chagas parasite, that there is no test that shows when it has disappeared, so you can take the treatment, eliminate the parasite (…). If you have (visceral) damage, cardiac damage, although you don’t have parasites or if they are, as we say, ‘asleep’, the damage is there. (…) It is important for women to try and take it (treatment) so as not to transmit it to their children, and for men to try to take it before the parasite causes damage, so let’s try to eliminate it.” (Key informant, Interview 3)
	“I think that instead of going to a school, which is multicultural and multi-origin (…) if a Bolivian person goes to a multicultural school, the other students will say ‘So, you are from Bolivia and you are infected’, right? Then comes the nickname ‘chagasic’ (…), racism and bullying, right? (…) We need to be careful.” (Key informant, Interview 1)

## Discussion

This study extends the application of Andersen’s Behavioural Model to infectious diseases, and in particular to CD, by proposing that the decision to know the diagnosis is a health behaviour influenced by various factors that exert more or less influence based on each person’s individual context. Additionally, the study adds stigma, family history, symptoms, and attitudes towards CD as additional contributions to the model. Andersen’s Behavioural Model has never been used for CD before. Previously, it has been mainly applied for HIV [38, 39].

The lack of knowledge and health beliefs about CD showed by young participants and men generates a prototypical speech in which, due to their migration, they no longer identify themselves with the profile of people who has CD (living in rural environments, being poor peasant in contact with the vector). This fact, together with the lack of knowledge about vertical transmission, is a very relevant aspect in non-endemic countries and is also very worrisome, because it could produce a feeling of invulnerability in non-endemic regions. In fact, previous studies showed a low knowledge about CD transmission, signs and symptoms, diagnosis and treatment in Bolivian population in non-endemic countries [5, 18, 19, 40–42]. In addition, diagnosis and treatment hesitancy in children is also a relevant aspect because it is known that this population group has the best treatment outcomes and fewer side effects [1].

On the other hand, despite them being people who have lived in Spain for many years, their working conditions remain precarious, so they have adapted their use of the health services to their needs as workers (for example, by using private health instead of public health services) [43–45]. Therefore, it seems that having a public health insurance card or having the right to health care is not the only element to take into consideration when evaluating access to CD diagnosis, since there are socioeconomic circumstances that condition equality in health [43]. In fact, Lalonde pointed out that healthcare system had less influence in health than other factors such as lifestyle or environmental factors [46].

In addition, although the administrative status of most of the participants is fairly regular, this population continues feeling rejected by Spanish society, and is very aware of their immigrant status [5, 42].

Most of participants were diagnosed as a consequence of pregnancy, blood donation and a positive diagnosis of a relative, whereas few of them decided to know their serologic status on their own. Therefore, it seems that the factors that most influence the decision to carry out the diagnosis for CD are having a relative with a positive diagnosis or deceased because of its complications, and, in the case of men, the coincidental finding of a positive diagnosis in a pregnant woman in the family [10, 47]. Furthermore, advice and support from family members and health professionals plays an important role for young people. Symptoms are not very relevant, either because of their absence or because of how they can be associated to their lifestyle.

Regarding strategies to facilitate CD diagnosis, these are mainly focused on physical access to the diagnostic test through information campaigns in key places and dates, timely screening from the usual health services, information focused on the prevention of complications, and on avoiding vertical transmission. These strategies are focused on improving factors such as population’s knowledge, accessibility to health services, and knowledge and predisposition of health providers, all of which need improving, as existing studies emphasize [5, 47–50]. However, support and social, family, or professional companionship must also be considered when designing strategies, since the fear of being sick or getting worse after knowing their diagnosis, and their conception of survival to CD are factors that also influence health behaviour, and have been found in previous research [21, 51]. Primary care physicians and nurses approach health in a holistic way, considering social determinants when promoting health and preventing possible disease. This, together with their proximity to the community and its resources, could help with supporting this population and their participation in strategies to modify health behaviours according to individual characteristics [52, 53].

From this point of view, some strategies have been developed in non-endemic countries with different results. Screening sessions and treatment were offered in Bergamo (Italy) with health education and counselling through cultural mediators and the involvement of the Latin American Community, although only 30% of targeted population completed treatment due to the centralization of diagnosis and treatment to the city [54]. In Spain, the community interventions developed and evaluated in Barcelona (Catalonia) in collaboration with associations, multidisciplinary teams, communication networks and health services promoted access to diagnosis, medical attention and social integration [55–57]. Social and family support and advice, subjective assessment of health and aspects linked to culture and health have been approached through promotion of paediatric awareness and active case searches of children born from pregnant women with CD, the Expert Patient Programme for Chagas Disease, workshops, community events and in situ screening [55, 57, 58].

Ideally, the design of strategies to improve the diagnosis of CD should influence each and every one of the factors identified in the model to achieve a change in the health behaviours of the Bolivian population in non-endemic regions, achieving a fluid relationship between patient, community and health system.

Being this study conducted in Madrid, these findings could not be generalizable to other contexts, although the results provide information that could improve healthcare of CD in non-endemic areas.

On the other hand, the sample is made up mostly of people with positive diagnosis, with their own experiences and those of their closest environment; therefore, it is likely that there is other population who has more difficulties with health system accessibility, whose health behaviours we do not know. Moreover, men were more reticent to participate in interviews, FGs and TGs than women, so their recruitment was difficult. There are two participants with unknown diagnosis whose testimony was included because of the information they provided about young Bolivian people and migration status. Despite these limitations, this study addresses in depth health behaviours of men and young population that provide relationships between different factors associated with the use of health services for the diagnosis of CD.

## Conclusions

The most important factors that influence the decision to carry out the diagnosis for CD are having a relative with a positive diagnosis or deceased because of its complications, and a positive diagnosis in a pregnant woman in the family. Other factors such as socioeconomic conditions, knowledge and attitudes about CD, stigma and healthcare environment hinder or facilitate the decision and the access to the diagnostic test. The results obtained in the present study regarding the decision of the population to carry out the diagnosis based on the Andersen’s Behavioural Model provide information that could help to develop strategies to improve access and modify health behaviours related to Chagas disease screening.

## Supporting information

S1 File Topic guide. https://doi.org/10.6084/m9.figshare.14226710.v2.
(DOCX)

S2 File Focus group/triangular group summary template. https://doi.org/10.6084/m9.figshare.14226710.v2.
(DOCX)

S3 File Interview summary template. https://doi.org/10.6084/m9.figshare.14226710.v2.
(DOCX)
