# Supplementary material for: Mapping health behaviour related to Chagas diagnosis in a non-endemic country: Application of Andersen’s Behavioural Model
Source: PLoS One. 2022 Jan 20;17(1):e0262772. doi: 10.1371/journal.pone.0262772 (PMC8775331; doi:10.1371/journal.pone.0262772)
Supplement: S1 File — https://doi.org/10.6084/m9.figshare.14226710.v2. (DOCX) [file pone.0262772.s001.docx]

| **TOPIC GUIDE** | |
| --- | --- |
| **THEMATIC RESEARCH QUESTIONS** | **DYNAMIC INTERVIEW QUESTIONS** |
| **PART 1. Knowledge about different aspects of Chagas disease: transmission routes, symptoms, treatment…** | To begin with, I would like to talk about Chagas disease. What can you tell me about Chagas disease? |
| Is the vector identified? Are the different routes of transmission known? | Do you know which is the bug that transmits Chagas? Have you ever seen it in Latin America? |
|  | Do you know another route of transmission apart from vinchuca’s bite? |
|  | Would you say that you could have got the disease in the past? |
| Are the different phases of Chagas disease identified, including asymptomatic phase? | What happens when you have the disease? Which are the symptoms of Chagas disease? Could Chagas cause death? |
| Is treatment for Chagas disease known? | Is there any treatment for Chagas? Which is your opinion about it? |
|  | Do you think is possible to get Chagas diagnosis and treatment in Spain? |
| **PART 2. Attitudes towards Chagas disease: stigma, need for medical attention, proclivity to use the health system…** | I would like to talk about the consequences of Chagas disease, the Spanish health system and the difficulties for diagnosis and treatment |
| Which are the consequences of Chagas disease? (labor, familiar, social) | Would you say that there are consequences on your job after getting Chagas disease? What about social life? And what about the consequences on your family? |
|  | Do you think there is a stigma associated to Chagas disease? |
| What are the effects of the fact that the disease is not endemic in Spain and only affects the immigrant population? | About that stigma, what is the relation between Chagas disease and being an immigrant? |
|  | Would you say that Spanish population knows the disease? Does that affect in any way? |
| Perceived need of medical attention in case of thinking about a possible contagion in the past | How is it necessary to receive medical attention if you think you may have Chagas disease? |
| What aspects of Spanish health system condition target population’s health services access? | Leaving Chagas disease aside for now, what would you say are your main problems when accessing the health system in Spain: the schedules, the distance, the treatment of personnel, financial resources, the health insurance card...? |
|  | How would you assess the quality of the health services you have received in Spain? Do you feel confident? |
| What aspects of the Spanish health system condition access to health services when diagnosing and treating Chagas disease? | And in the particular case of Chagas disease, does the Spanish health system give you confidence to diagnose and treat the disease? Would you go to the health system if you thought you might have Chagas disease? |
|  | How would you assess the level of information of Spanish medical professionals about Chagas disease? |
| **PART 3. Practices in relation to Chagas disease: care itineraries for the diagnostic test, treatment, prevention strategies and management of the disease ...** | I would like to talk about the way in which you got the Chagas disease test and what problems you encountered along that path. |
| What has been the healthcare itinerary that has allowed access and use of the health system to carry out the diagnostic test for Chagas disease? | I wanted you to tell me about your decision to take the diagnostic test. Did someone advise you? Who? Where did you go to get the diagnostic test? |
|  | Did you find any difficulty or problem when you got the diagnostic test? |
| What is the opinion about the treatment and in what way does it affect the different spheres of the subject's life? | Have you received treatment for Chagas disease? What is your opinion about the treatment? Have you ever interrupted it? What reasons led you to interrupt it? Which aspects in your life have been most affected by the treatment? |
| What practices are carried out in order to avoid the spread of Chagas disease and to live with the disease? | Give me an example of practices that you carry out to prevent the spread of the disease / Give me an example of practices that you carry out to live with Chagas disease |
| **PART 4. Strategies that could facilitate Chagas disease diagnosis** | Finally, I would like you to tell me what things would make it easier to take the test. |
