# Supplementary material for: Mapping health behaviour related to Chagas diagnosis in a non-endemic country: Application of Andersen’s Behavioural Model
Source: PLoS One. 2022 Jan 20;17(1):e0262772. doi: 10.1371/journal.pone.0262772 (PMC8775331; doi:10.1371/journal.pone.0262772)
Supplement: S2 File — https://doi.org/10.6084/m9.figshare.14226710.v2. (DOCX) [file pone.0262772.s002.docx]

**POPULATION’S FOCUS GROUP/TRIANGULAR GROUP SUMMARY TEMPLATE**

Contact the informants and agree on the place, day and time of the group. Prepare the group script and the materials needed for its development, check:

- Recorders and batteries.
- Copies of the study and informed consent for each participant.
- Ballpoint pen.
- Water.
- Glasses.
- Cardboards.
- Chairs.
- Working documents of the moderator and the observer.
- Don’t forget to do a backup copy of the recording.

| **Focus/triangular group:** | | | | | | | | | |
| --- | --- | --- | --- | --- | --- | --- | --- | --- | --- |
| Number of the focus/triangular group: | | | | | Weekday: | | | | |
| Place: | | | | | Date and time: | | | | |
| Moderator: | | | | | Observer: | | | | |
| Duration: | | | | | Recording: | | | | |
| Informed consent: | | | | | Contact person: | | | | |
| Diagram of the participants and description of the physical environment (objects and people) | | | | | | | | | |
| Participants and their characteristics (annotations according to their presentations): | | | | | | | | | |
| N.º | Age | Sex | Region of origin | Education level | | Employment status | Public health coverage | Nº of children | Years in Spain |
| P1 |  |  |  |  | |  |  |  |  |
| P2 |  |  |  |  | |  |  |  |  |
| P3 |  |  |  |  | |  |  |  |  |
| P4 |  |  |  |  | |  |  |  |  |
| P5 |  |  |  |  | |  |  |  |  |
| P6 |  |  |  |  | |  |  |  |  |
| P7 |  |  |  |  | |  |  |  |  |
| P8 |  |  |  |  | |  |  |  |  |
| P9 |  |  |  |  | |  |  |  |  |
| Annotations (use the space that is necessary): | | | | | | | | | |
| Content of the focus/triangular group group (maximum two pages): | | | | | | | | | |
