# Supplementary material for: Mapping health behaviour related to Chagas diagnosis in a non-endemic country: Application of Andersen’s Behavioural Model
Source: PLoS One. 2022 Jan 20;17(1):e0262772. doi: 10.1371/journal.pone.0262772 (PMC8775331; doi:10.1371/journal.pone.0262772)
Supplement: S3 File — https://doi.org/10.6084/m9.figshare.14226710.v2. (DOCX) [file pone.0262772.s003.docx]

**POPULATION’S INTERVIEW SUMMARY TEMPLATE**

Contact the informant and agree on the place, day and time of the interview. Prepare the interview script and the materials needed for its development, check:

- Recorders and batteries.
- Copies of the study and informed consent.
- Ballpoint pen.
- Working documents of the interviewer.
- Don’t forget to do a backup copy of the recording.

| **Interview** | | | | | | | | |
| --- | --- | --- | --- | --- | --- | --- | --- | --- |
| N.º | Age | Sex | Region of origin | Education level | Employment status | Public health coverage | Nº of children | Years in Spain |
|  |  |  |  |  |  |  |  |  |
| Number of the interview: | | | | | | | | |
| Place: | | | | | | | | |
| Date and time: | | | | | | | | |
| Interviewer: | | | | | | | | |
| Duration: | | | | | | | | |
| Contact person: | | | | | | | | |
| Recording: | | | | | | | | |
| Informed consent: | | | | | | | | |
| Participants and their characteristics (annotations according to their presentations): | | | | | | | | |
| Annotations (use the space that is necessary): | | | | | | | | |
